# PRMT5-Mediated ALKBH5 Methylation Promotes Colorectal Cancer Immune Evasion via Increasing CD276 Expression

**DOI:** 10.34133/research.0549

**Published:** 2025-01-08

**Authors:** Sen Meng, Hao Liu, Jiayu Xu, Chuyin Deng, Xingyou Qian, Sufang Chu, Wei-Guo Zhu, Jiuling Zhu, Hongmei Yong, Zhongwei Li, Jin Bai

**Affiliations:** ^1^ Cancer Institute, Xuzhou Medical University, Xuzhou, Jiangsu, China.; ^2^ Centre of Clinical Oncology, The Affiliated Hospital of Xuzhou Medical University, Xuzhou, Jiangsu, China.; ^3^ Department of Oncology, The Affiliated Huai’an Hospital of Xuzhou Medical University and The Second People’s Hospital of Huai’an, Huai’an, Jiangsu, China.; ^4^ Laboratory of Epigenetic Regulation in Molecular Medicine, Department of Pathophysiology, School of Basic Medical Sciences, Wannan Medical College, Wuhu, Anhui, China.; ^5^ International Cancer Center, Guangdong Key Laboratory of Genome Instability and Human Disease Prevention, Marshall Laboratory of Biomedical Engineering, Department of Biochemistry and Molecular Biology, Shenzhen University Medical School, Shenzhen, China.; ^6^ Anhui Province Key Laboratory of Basic Research and Transformation of Age-Related Diseases, Wannan Medical College, Wuhu, Anhui, China.; ^7^ Jiangsu Center for the Collaboration and Innovation of Cancer Biotherapy, Cancer Institute, Xuzhou Medical University, Xuzhou, Jiangsu, China.

## Abstract

Numerous diseases have been connected to protein arginine methylations mediated by protein arginine methyltransferase 5 (PRMT5). Clinical investigations of the PRMT5-specific inhibitor GSK3326595 are currently being conducted, and the results are promising for preventing cancers. However, the detailed mechanism of PRMT5 promoting colorectal cancer (CRC) malignant progression remains unclear. Here, we found that PRMT5 directly catalyzes AlkB homologue 5 (ALKBH5) symmetric dimethylation at the R316 residue (meR316-ALKBH5), which enhances TRIM28-mediated ALKBH5 ubiquitination degradation. Then, an ALKBH5 decrease attenuates ALKBH5-mediated m6A demethylation on the CD276 transcript 3′ untranslated region, which increases CD276 messenger RNA stability and its expression in CRC cells. Furthermore, a CD276 expression increase facilitates CRC immune evasion by inhibiting cytotoxic T-cell functions. Moreover, we revealed that PRMT5-mediated meR316-ALKBH5 activates CD276 transcription by increasing its messenger RNA m6A modification to increase CRC immune evasion in vitro and in vivo. Furthermore, we consistently showed a strong association between meR316-ALKBH5 and poor outcomes in patients with CRC. Finally, we demonstrated that combining an anti-PD1 antibody with the PRMT5 inhibitor GSK3326595 markedly halts the progression of CRC. Our findings could serve as a basis for the development of a PRMT5–meR316-ALKBH5–CD276 axis-targeting treatment approach for CRC.

## Introduction

Colorectal cancer (CRC) is the third most common malignancy and the third leading cause of cancer-related deaths worldwide [1–4]. It is pressing to explore the underlying molecular processes and develop innovative treatment methods targeting novel targets to prevent the progression of CRC in patients.

Protein methylation, a posttranslational modification (PTM), takes place on specific lysine or arginine residues localized in both histone and nonhistone proteins [5]. This alteration allows the targeted protein to have distinct features in a variety of disorders [6–8]. Protein arginine methylation is one common but understudied PTM found on nuclear and cytoplasmic proteins associated with cancer progression [9–11]. According to our most recent research, the methylation of arginine is necessary for several biological functions, including cell senescence [12], oxidative phosphorylation [13], and tolerance to oxidative stress [14,15], as well as cancer metastasis [16–20].

Protein arginine methyltransferase 5 (PRMT5) is the major methyltransferase that catalyzes symmetric dimethylation (SDMA) and profoundly leads to numerous biological mechanisms, such as epigenetic regulation of transcription [21–23], splicing regulation [24], circadian rhythm regulation [25], response to DNA damage, and germ cell formation [26,27]. Recent studies have shown that improved antitumor immunity is correlated with a greater quantity of infiltrating immune cells after PRMT5 suppression [28,29]. In this way, it is anticipated that PRMT5 inhibition will improve the way that immunological checkpoint treatment reacts to unresponsive cancers [30,31]. GSK3326595 (short for GSK595 in figures), a very potent and highly specific small-molecule inhibitor, binds to the catalytic site of PRMT5. This inhibits PRMT5 methyltransferase activity, which decreases the level of SDMA modifications on downstream targets [32]. The newest clinical trials in phase I/II have indicated that GSK3326595 exhibits notable anticancer and favorable prognostic effects against a variety of solid tumors and carcinomas (NCT02783300 and NCT03614728). We sought to determine the pathway by which PRMT5 restricts the activation and recruitment of immune cells, which defines tumor immune evasion, as well as the therapeutic effect of GSK3326595 in CRC.

In this study, we showed that SDMA of PRMT5-mediated human AlkB homologue 5 (ALKBH5, an m6A demethylase) weakens global m6A levels in CRC cells. We demonstrated that the ALKBH5-R316 SDMA (meR316-ALKBH5) modification mediated by PRMT5 facilitates the binding of TRIM28 to ALKBH5, resulting in the ubiquitination-mediated degradation of ALKBH5. We demonstrated that PRMT5-mediated meR316-ALKBH5 promotes CD276 messenger RNA (mRNA) stability through m6A modification, thereby enhancing CRC tumor immune evasion both in vitro and in vivo. Moreover, we found a significant correlation between meR316-ALKBH5 modification and poor prognosis in CRC patients. Furthermore, we reported that combination therapy with GSK3326595 and anti-PD1 antibodies significantly inhibited the progression of CRC. Our results indicate that GSK3326595, a PRMT5-specific inhibitor, is a potentially effective drug for reducing CRC immune evasion by blocking ALKBH5 methylation and ALKBH5–CD276 interaction.

## Results

### PRMT5 depletion decreases the global m6A level in CRC cells

**Fig. 1.**
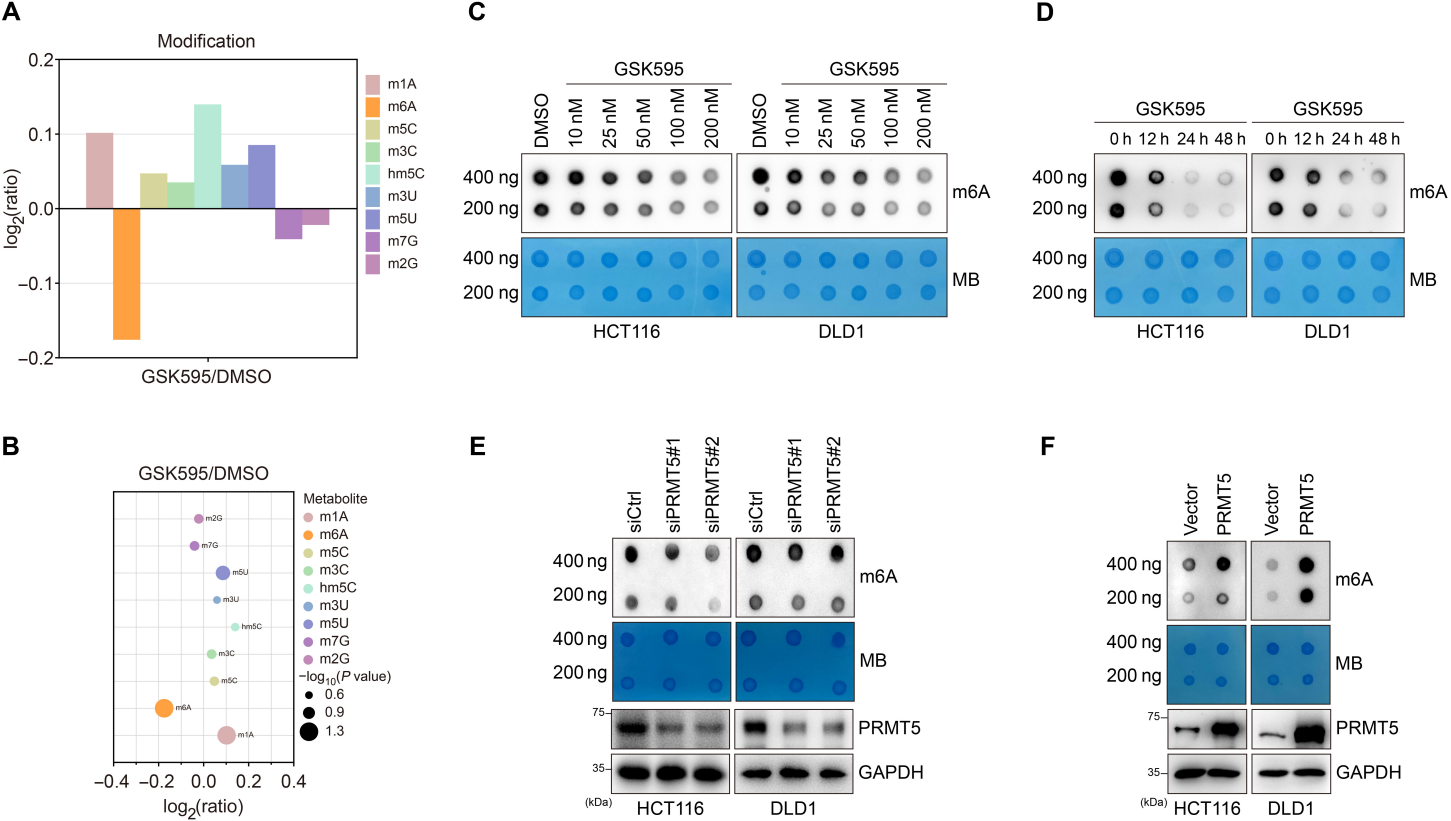
Protein arginine methyltransferase 5 (PRMT5) inhibitor or knockdown reduces the global level of m6A methylation modification in colorectal cancer cells. (A) Modified nucleosides of colorectal cancer cells treated with GSK595 or DMSO were quantified using liquid chromatography coupled with tandem mass spectrometry (LC–MS/MS). The relative amounts of modified nucleosides in the comparative group (GSK595/DMSO) are shown in bar plots. log_2_(ratio) was taken to symmetrize the ratio; log_2_(ratio) > 0 indicates that the nucleoside is up-regulated in the comparison group, and log_2_(ratio) < 0 indicates that the nucleoside is down-regulated in the comparison group. The m6A level intensely declined. (B) Scatter plots displaying the change in each modified nucleoside in GSK595/DMSO; m6A modification shows significant down-regulation. (C and D) Dot-blot assay of RNA m6A methylation in HCT116 and DLD1 colorectal cancer cells treated with GSK595 for the dose and duration indicated. (E and F) Dot-blot assay of RNA m6A methylation in HCT116 and DLD1 colorectal cancer cells down-regulating or overexpressing PRMT5. GAPDH, glyceraldehyde-3-phosphate dehydrogenase.

Evidently, protein arginine methyltransferase inhibitors can increase the susceptibility of cancer cells to anticancer therapy. However, the mechanism by which PRMT5 inhibition overcomes anticancer drug resistance is unknown. Nucleoside modifications (especially m6A modifications) have been reported to play a key role in cancer progression [33]. For this purpose, we examined the changes in nucleoside modifications in CRC cells treated with GSK3326595 by liquid chromatography–mass spectrometry (MS) (Tables S1 to S3). Compared to other nucleoside modifications, GSK3326595 dramatically decreased the N6-methylation of adenosine (m6A) level in CRC cells (Fig. 1A), and we discovered that the m6A modification ratio was dramatically decreased (Fig. 1B). In addition, we calculated the ratio changes between nucleoside modifications and the corresponding nucleosides, and the ratio of m6A to A was the lowest (Fig. S1A and B).

We used a dot-blot assay to determine the effect of GSK3326595 on RNA m6A in CRC cells. GSK3326595 induced a significant decrease in RNA m6A methylation in a time- and dose-dependent manner (Fig. 1C and D). Furthermore, we found that the down-regulation of PRMT5 reduced the m6A level (Fig. 1E), while the overexpression of PRMT5 increased the m6A level in HCT116 and DLD1 cells (Fig. 1F). These findings showed that a reduction in PRMT5 enzyme activity or expression reduces the global level of m6A modification in CRC cells.

### PRMT5 decreases ALKBH5 stability and expression by increasing its ubiquitination-mediated degradation

**Fig. 2.**
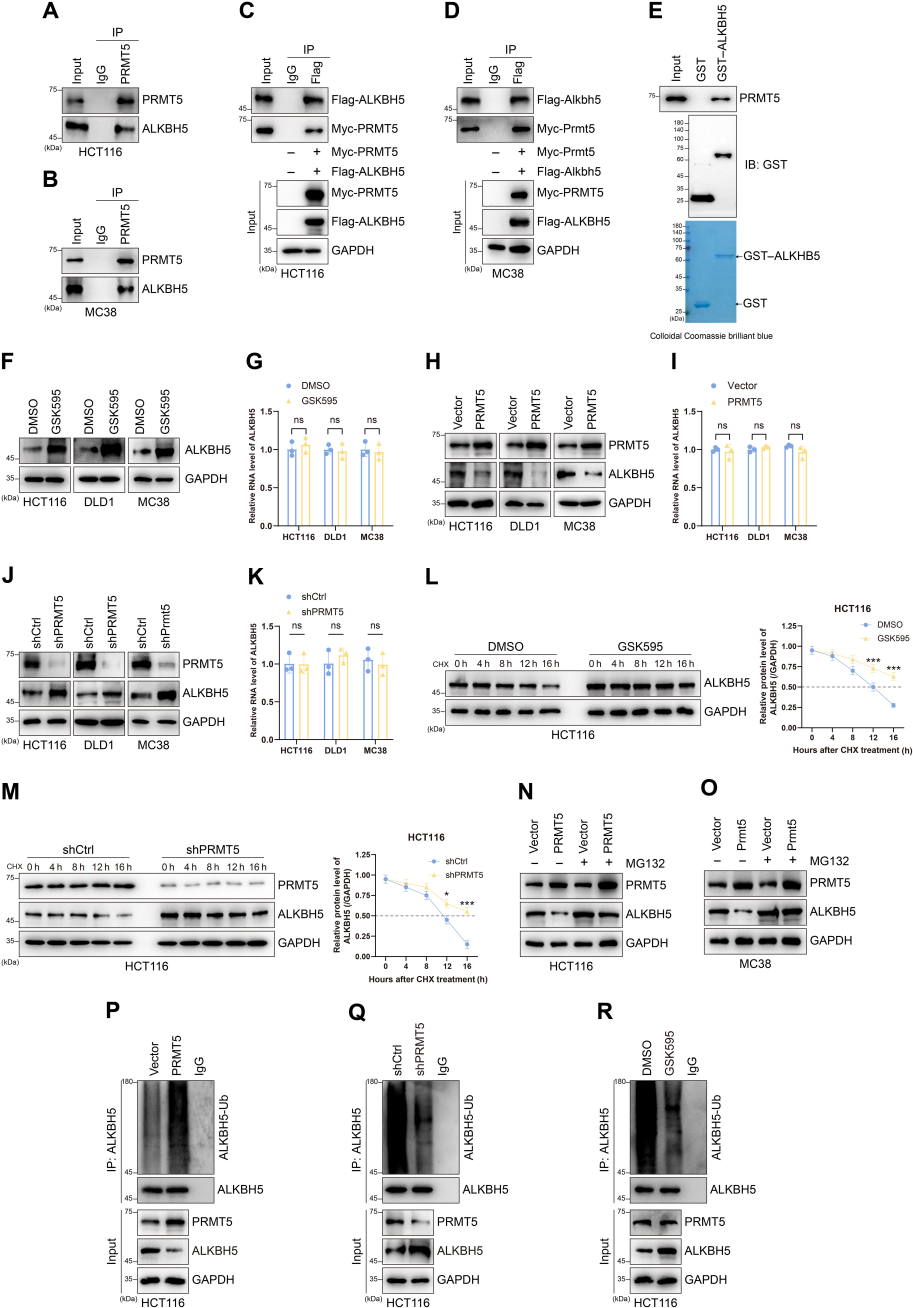
PRMT5 weakens AlkB homologue 5 (ALKBH5) stability by increasing ALKBH5 ubiquitination. (A and B) Cell lysates from HCT116 or MC38 cells were immunoprecipitated with antibodies against indicated proteins followed by immunoblotting with various antibodies indicated. (C and D) Flag-ALKBH5 and Myc-PRMT5, Flag-Alkbh5, and Myc-Prmt5 were coexpressed in HCT116 and MC38 cells, respectively. After immunoprecipitation with appropriate antibodies, bound proteins were examined by Western blotting. (E) Glutathione-*S*-transferase (GST)-fused ALKBH5 was incubated with HEK293T cell lysates. The binding proteins by GST pull-down assays were examined by Western blotting with the indicated antibodies. The amounts of GST and GST-tagged ALKBH5 were visualized by Coomassie blue staining. (F and G) ALKBH5 protein and messenger RNA (mRNA) levels were analyzed by Western blot and quantitative reverse transcription polymerase chain reaction (qRT-PCR) experiments in HCT116, DLD1, and MC38 cells treated by the PRMT5 inhibitor GSK595. (H and I) Protein and mRNA expression of ALKBH5 were assessed by Western blot and qRT-PCR assays after overexpression PRMT5. (J and K) ALKBH5 proteins and mRNAs were detected by Western blot and qRT-PCR after knockdown of PRMT5. (L and M) The effect of PRMT5 pharmacological (GSK595) or genetic (PRMT5 knockdown) inhibition on ALKBH5 protein expression was detected by Western blot in HCT116 cells following treatment by CHX (50 μg/ml) for the indicated time (left). The relative intensities of ALKBH5 proteins were quantified by the software ImageJ (right). For normalization, GAPDH expression was used as a control. *P < 0.05; ***P < 0.001. (N) Western blot showing the effects of proteasome inhibitor MG132 (10 μM for 8 h) treatment on ALKBH5 protein accumulation in HCT116 cells. (O) Western blot showing effects of proteasome inhibitor MG132 (10 μM for 8 h) treatment on ALKBH5 protein accumulation in MC38 cells. (P) PRMT5 was overexpressed in HCT116 cells, and immunoprecipitation was performed using an anti-ALKBH5 antibody, which suggested that PRMT5 could bind to ALKBH5 and increase the ubiquitination degradation level of ALKBH5. (Q and R) PRMT5 was pharmacologically (GSK595) or genetically inhibited (PRMT5 knockdown) in HCT116 cells, and immunoprecipitation was performed using an anti-ALKBH5 antibody, which suggested that PRMT5 could decrease ALKBH5 ubiquitination degradation. IP, immunoprecipitation; IgG, immunoglobulin G; ns, not significant.

To determine the mechanism by which PRMT5 regulates m6A modification in CRC cells, we used MS analysis to explore the downstream effectors of PRMT5. MS analysis indicated that the PRMT5 immunoprecipitates contained ALKBH5 (Fig. S2A). Through Western blotting, the presence of ALKBH5 in the PRMT5 interactome was confirmed (Fig. S2B). We used both endogenous and exogenous proteins in our coimmunoprecipitation (Co-IP) studies to examine whether ALKBH5 is a bona fide interactor of PRMT5. We discovered that the immunoprecipitation (IP) of endogenous PRMT5 from HCT116 and MC38 cells decreased the level of ALKBH5 (Fig. 2A and B). Moreover, tagged PRMT5 and ALKBH5 plasmids were concurrently transferred into CRC cells, and Co-IP experiments were conducted to determine whether PRMT5 and ALKBH5 interacted. The results demonstrated that PRMT5 and ALKBH5 can bind to one another (Fig. 2C and D). Glutathione-*S*-transferase (GST) pull-down confirmed these findings and provided additional evidence for interactions between PRMT5 and ALKBH5 (Fig. 2E). These results collectively demonstrate that PRMT5 specifically interacts with ALKBH5 both in vivo and in vitro.

We further explored changes in key metabolic enzymes involved in m6A modification, including the m6A methyltransferases METTL3, METTL14, and METTL16 and the m6A demethyltransferases FTO and ALKBH5, in HCT116, DLD1, and MC38 cells treated with GSK3326595. We found that GSK3326595 did not affect the expression of METTL3, METTL14, METTL16, or FTO (Fig. S2C), while GSK3326595 down-regulated the expression of ALKBH5 (Fig. 2F). However, the ALKBH5 mRNA levels exhibited minimal variation in CRC cells (Fig. 2G). Next, we demonstrated that the overexpression of PRMT5 resulted in a decrease in ALKBH5; however, there were no alterations in the ALKBH5 transcriptional level (Fig. 2H and I). Conversely, down-regulated PRMT5 improved ALKBH5 expression in CRC cells, but the changes in ALKBH5 mRNA expression were not statistically significant (Fig. 2J and K).

We hypothesized that PRMT5 influences ALKBH5 stability via the ubiquitin–proteasome pathway. GSK3326595 prolonged the half-life of ALKBH5 in HCT116 and MC38 cells after treatment with CHX (Fig. 2L and Fig. S2D). We used HCT116 and MC38 cells to generate shPRMT5 and shCtrl stable cell lines, respectively. Consistently, we found that the half-life of ALKBH5 was much longer in HCT116 and MC38 shPRMT5 cells treated with CHX (Fig. 2M and Fig. S2E). Additionally, we administered the proteasome inhibitor MG132 to vector-transfected or PRMT5-overexpressing cells. MG132 prevented the reduction in ALKBH5 expression induced by PRMT5 overexpression in HCT116 and MC38 cells (Fig. 2N and O). Furthermore, IP analysis revealed that PRMT5 overexpression enhanced the ubiquitination-mediated degradation of ALKBH5 in HCT116 cells (Fig. 2P). However, the application of GSK3326595 or a reduction in PRMT5 expression in CRC cells reduced the degradation of ubiquitinated ALKBH5 (Fig. 2Q and R). Taken together, these findings support our hypothesis that PRMT5 weakens ALKBH5 stability by increasing ALKBH5 ubiquitination.

### PRMT5-mediated ALKBH5-R316 methylation strengthens TRIM28-mediated ALKBH5 ubiquitination-mediated degradation

**Fig. 3.**
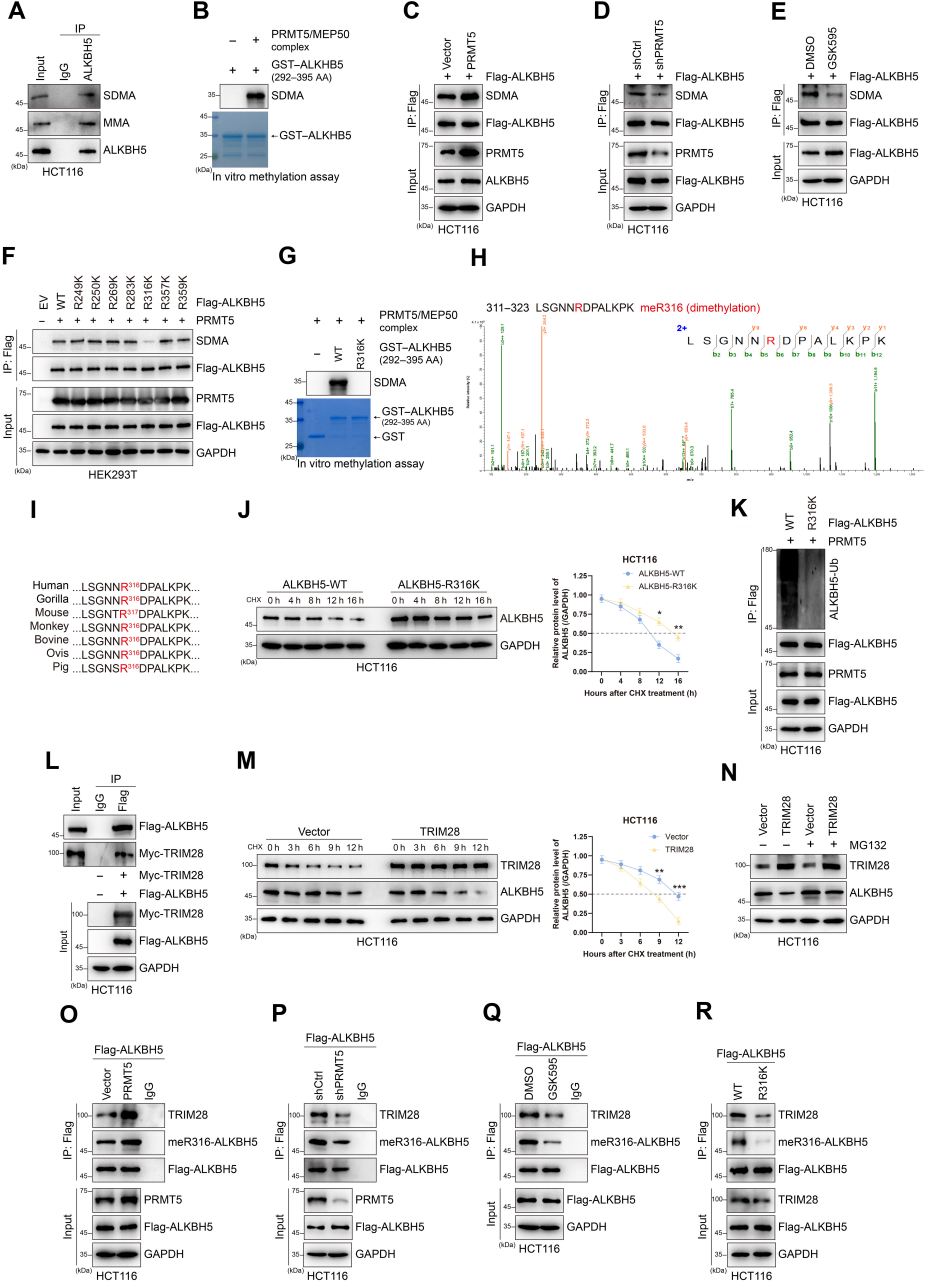
PRMT5-mediated meR316-ALKBH5 decreases ALKBH5 stability by TRIM28-mediated ALKBH5 degradation. (A) Symmetric dimethylation (SDMA) and MMA of endogenous ALKBH5 in HCT116 cells were assessed by IP assays. (B) In vitro methylation assays. Purified GST-tag fusion proteins of GST–ALKBH5 (292 to 395 AA) were incubated with the PRMT5/MEP50 complex. Methylated proteins were detected via immunoblotting, and the total amounts of proteins were visualized by Coomassie blue staining (arrows: position of GST–ALKBH5 [292 to 395 AA]). (C) PRMT5 and Flag-ALKBH5 were overexpressed in HCT116 cells simultaneously, and IP was performed using an anti-Flag antibody, which suggested that PRMT5 could increase the SDMA level of ALKBH5. (D) Detection of ALKBH5 SDMA modification status after IP Flag-ALKBH5 in PRMT5 down-regulated HCT116 cells. (E) Detection of ALKBH5 SDMA modification status after IP Flag-ALKBH5 in HCT116 cells treated with GSK595. (F) In vivo methylation detection of SDMA-ALKBH5 levels in HEK293T cells overexpression of wild-type (WT) ALKBH5 and R249K/R250K/R269K/R283K/R316K/R357K/R359K mutant ALKBH5, respectively. (G) Purified GST-tag fusion proteins of WT and R316K GST–ALKBH5 (292 to 395 AA) were incubated with the PRMT5/MEP50 complex in in vitro methylation assays. Methylated proteins were detected via immunoblotting, and the total amounts of proteins were visualized by Coomassie blue staining. (H) Mass spectrometry (MS) analysis of ALKBH5 methylation. The fragmentation of the ALKBH5 peptide LSGNNRDPALKPK identified a dimethylated residue at R316. (I) Sequence alignments of ALKBH5 in mammals. The ALKBH5-R316 site is denoted in the protein sequences. (J) The ALKBH5 expression was detected by Western blotting after expression of ALKBH5-WT or ALKBH5-R316K in HCT116 cells treated with CHX (50 μg/ml). The bands of ALKBH5 proteins treated by CHX were quantified by the software ImageJ. *P < 0.05; **P < 0.01. (K) Western blots of Flag-ALKBH5-associated ubiquitination after IP ALKBH5-Ub in HCT116-ALKBH5-WT cells and HCT116-ALKBH5-R316K cells. (L) Western blot analysis of exogenous interaction between ALKBH5 and TRIM28 after IP Tagged-ALKBH5 in HCT116 cells. (M) TRIM28 overexpression decreased ALKBH5 protein half-life. CHX (50 μg/ml) was added in HCT116 and TRIM28-overexpressing HCT116 cells at the indicated time points. Cell lysates were then subjected to immunoblotting analyses. **P < 0.01; ***P < 0.001. (N) Western blot showing effects of proteasome inhibitor MG132 (10 μM for 8 h) treatment on ALKBH5 protein accumulation in HCT116 cells. (O) Western blots of meR316-ALKBH5 and TRIM28 binding with Flag-ALKBH5 after IP Flag-ALKBH5 in Flag-ALKBH5- and PRMT5-coexpressing HCT116 cells compared to HCT116 Flag-ALKBH5 cells. (P) Western blots of meR316-ALKBH5 and TRIM28 interaction with ALKBH5 in shCtrl and shPRMT5 cells after IP Flag-ALKBH5. (Q) Western blots detected meR316-ALKBH5 and TRIM28 interacting with ALKBH5 after coimmunoprecipitation (Co-IP) Flag-ALKBH5 from HCT116 cells treated by PRMT5 inhibitor GSK595. (R) Western blot analysis of meR316-ALKBH5 and TRIM28 binding with ALKBH5 after IP Flag-ALKBH5 in HCT116-ALKBH5-WT cells and HCT116-ALKBH5-R316K cells. AA, amino acids.

Our previous studies showed that arginine methylation of EZH2 can regulate its protein stability [18]. Combined with our findings in this study, we wondered whether PRMT5-mediated ALKBH5 methylation results in decreased protein stability. To explore the possibility of arginine methylation of ALKBH5, IP experiments were carried out in CRC cells. We found that endogenous ALKBH5 underwent SDMA and MMA modification (Fig. 3A and Fig. S3A). Importantly, in vitro methylation tests revealed that PRMT5 directly mediates SDMA-ALKBH5 production (Fig. 3B). In addition, PRMT5 overexpression promoted the modification of ALKBH5 by SDMA in HCT116 cells, as shown by IP experiments (Fig. 3C). Conversely, knockdown of PRMT5 or treatment with GSK3326595 in HCT116 cells reduced the level of the SDMA modification of ALKBH5 (Fig. 3D and E). These results verify that PRMT5 is able to regulate the SDMA modification of ALKBH5.

Moreover, our methylation MS analysis of the methylated ALKBH5 protein revealed that the arginine residues at 249, 250, 269, 283, 316, 357, and 359 were potentially methylated by PRMT5 in vitro (Fig. S3B). To confirm the crucial ALKBH5 methylation sites, we individually altered the R249, R250, R269, R283, R316, R357, and R359 residues in Flag-ALKBH5 with lysine (K). Our in vivo methylation experiments revealed that the R316K mutation alone significantly reduced the PRMT5-mediated methylation of ALKBH5 (Fig. 3F). In vitro methylation assays revealed that mutant ALKBH5 in R316 cannot be methylated by PRMT5 (Fig. 3G). MS data indicated that R316 was dimethylated by PRMT5 (Fig. 3H), proving that PRMT5 mainly dimethylates ALKBH5 at the R316 site. Since PRMT5 solely catalyzes the production of arginine residues in SDMA, the ALKBH5-R316 SDMA modification was mediated by PRMT5. The R316 methylation site (the R317 site in mice) on ALKBH5 is highly conserved in mammals (Fig. 3I). Next, we produced an antibody that precisely identified ALKBH5 SDMA at R316 (anti-meR316-ALKBH5) (Fig. S3C and D).

In addition, our half-life assays revealed that the half-life of the ALKBH5-R316K mutant was longer than that of ALKBH5-wild type (WT) in HCT116 and MC38 cells (Fig. 3J and Fig. S3E). We also found that the ALKBH5-R316K-overexpressing group had lower ALKBH5 ubiquitination than the ALKBH5-WT-overexpressing group in HCT116 and MC38 cells (Fig. 3K and Fig. S3F), which means that PRMT5 exclusively affects the protein stability of ALKBH5-WT but not that of ALKBH5-R316K. Taken together, these findings suggest that R316-ALKBH5 methylation via PRMT5 is essential for maintaining ALKBH5 stability.

Accordingly, we sought to identify the particular E3 ligase involved in the ability of PRMT5-mediated meR316-ALKBH5 to promote ALKBH5 ubiquitination. Our IP Flag-ALKBH5 MS data showed that ALKBH5 may bind to TRIM28, TRIM25, and USP14. In HCT116 and MC38 cells, we discovered that PRMT5 exclusively coimmunoprecipitated with TRIM28 but not with TRIM25 or USP14 (Fig. S3G and H). We subsequently verified that exogenous ALKBH5 and TRIM28 were able to bind to each other in HCT116 and MC38 cells (Fig. 3L and Fig. S3I).

After that, we focused on whether TRIM28 affects ALKBH5. Our data showed that when TRIM28 was overexpressed in HCT116 and MC38 cells, ALKBH5 expression decreased at the protein level (Fig. S3J). When TRIM28 was overexpressed, the ALKBH5 mRNA level did not significantly change (Fig. S3K). Additionally, our results showed that overexpression of TRIM28 shortened the half-life of ALKBH5 in HCT116 and MC38 cells treated with CHX (Fig. 3M and Fig. S3L). The reduction in ALKBH5 caused by TRIM28 overexpression in HCT116 and MC38 cells was prevented by the proteasome inhibitor MG132 (Fig. 3N and Fig. S3M). These findings suggest that TRIM28 may regulate ALKBH5 protein stability. We reasoned that TRIM28 might control the expression of ALKBH5 by functioning as an E3 ubiquitin ligase. IP revealed that the interaction of TRIM28 with ALKBH5 resulted in enhanced ubiquitination-mediated degradation of ALKBH5 (Fig. S3N). The above data strongly indicate that the E3 ligase TRIM28 directly mediates the ubiquitination and degradation of ALKBH5.

To further explore whether PRMT5-mediated meR316-ALKBH5 regulates TRIM28-mediated ALKBH5 ubiquitination-mediated degradation, we performed co-IP with Flag-ALKBH5 to determine the extent of the ALKBH5–TRIM28 interaction when Flag-ALKBH5 and PRMT5 were coexpressed compared to the extent to which Flag-ALKBH5 was overexpressed in HCT116 cells. Moreover, as demonstrated by co-IP, the binding of TRIM28 to Flag-ALKBH5 was greater in the Flag-ALKBH5- and PRMT5-coexpressing HCT116 cells than in the corresponding HCT116 Flag-ALKBH5 cells (Fig. 3O). Consequently, we conducted co-IP assays on HCT116-shCtrl cells, HCT116-shPRMT5 cells, and HCT116 cells treated with GSK595 or DMSO to determine the amount of TRIM28 that interacts with ALKBH5 and meR316-ALKBH5. According to our findings, PRMT5 knockdown and GSK595 treatment reduced the interaction of meR316-ALKBH5 and TRIM28 with ALKBH5 (Fig. 3P and Q). Then, we verified that the interactions of meR316-ALKBH5 and TRIM28 with ALKBH5 were significantly lower in the ALKBH5-R316K cells than in the ALKBH5-WT cells (Fig. 3R). HCT116 cells containing ALKBH5-WT were treated with the proteasome inhibitor MG132, the lysosome inhibitor leupeptin, or the neddylation inhibitor MLN4924, but only MG132 treatment reversed ALKBH5 expression (Fig. S3O), suggesting that the ubiquitin–proteasome pathway might be responsible for ALKBH5 degradation. Remarkably, arginine mutation to lysine (ALKBH5-R316K) strengthened the protein expression of ALKBH5 (Fig. S3O), suggesting that arginine methylation of ALKBH5 is essential for its protein stability. Taken together, these findings confirm that ALKBH5 ubiquitination is strengthened by PRMT5-mediated R316-ALKBH5 methylation, which decreases ALKBH5 stability.

### R316-ALKBH5 methylation is required for CRC cell immune evasion in vitro and in vivo

**Fig. 4.**
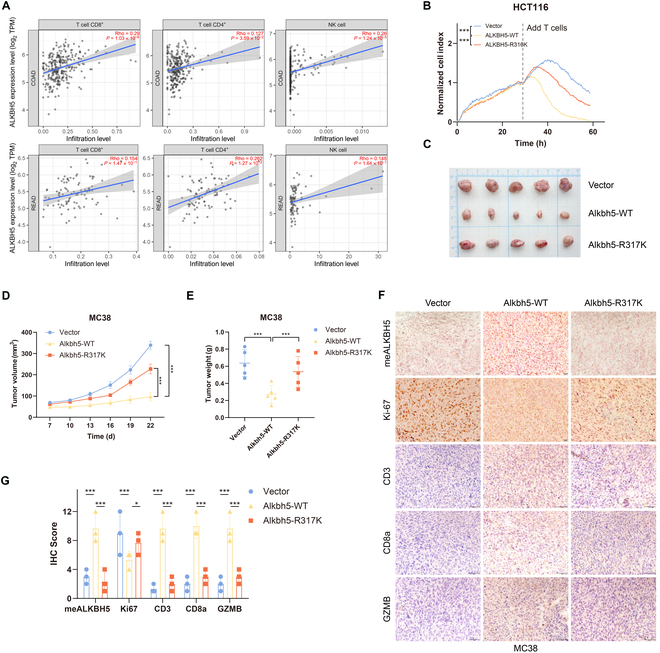
meR316-ALKBH5 plays a pivotal role in regulating colorectal cancer cells’ immune evasion in vitro and in vivo. (A) ALKBH5 expression positively associated with 3 kinds of tumor-infiltrating immune cells (TIICs) in TIMER2.0. (B) Effect of ALKBH5-WT or ALKBH5-R316K on HCT116 cells’ proliferation with T-cell killing as assessed by Real-Time Cell Analyzer (RTCA, xCELLigence) assays. (C) MC38 cells were injected into C57BL mice after the transfection of Alkbh5-WT, Alkbh5-R317K, and the vector respectively. (D and E) The growth curves and weights of tumors from subcutaneously injected C57BL mice treated with Alkbh5-WT, Alkbh5-R317K, and the vector separately. (F) Representative images of the immunohistochemistry (IHC) staining of methylated ALKBH5 (meALKBH5), Ki-67, CD3, CD8a, and GZMB in the tumor xenografts. (G) Quantification of meALKBH5, Ki-67, CD3, CD8a, and GZMB staining intensity in xenograft tumors. *P < 0.05; ***P < 0.001.

There is evidence that ALKBH5 can influence cancer immunity [34,35]. We aimed to gain more insight into the connection between the abundance of tumor-infiltrating immune cells (TIICs) and the expression level of ALKBH5 in CRC cells. The TIMER2.0 database was used to demonstrate a significant positive correlation of ALKBH5 with TIICs, including CD8^+^ T cells (*r* = 0.29, *P* =1.03 × 10^−6^), CD4^+^ T cells (*r* = 0.127, *P* = 3.59 × 10^−2^), and NK cells (*r* = 0.26, *P* = 1.24 × 10^−5^), in COAD (Fig. 4A). Similar relationships between ALKBH5 expression and CD8^+^ T cells (*r* = 0.154, *P* =1.47 × 10^−1^), CD4^+^ T cells (*r* = 0.262, *P* = 1.27 × 10^−2^), and NK cells (*r* = 0.148, *P* = 1.64 × 10^−1^) were detected in READ (Fig. 4A).

Accordingly, in vitro T-cell killing experiments were performed to investigate whether PRMT5-mediated R316-ALKBH5 methylation is necessary for impairing CRC cell immune evasion. We discovered that, compared with those of the ALKBH5-R316K and vector cells, the growth of HCT116 cells was significantly reduced only in the ALKBH5-WT cells (Fig. 4B).

We also evaluated the relationship between PRMT5-mediated ALKBH5 methylation and CRC cell immune evasion in vivo through a xenograft model. MC38-Alkbh5-WT cells or MC38-Alkbh5-R317K cells were subcutaneously implanted into C57BL mice. Cells expressing Alkbh5-WT formed smaller tumors (Fig. 4C), and the tumor growth curve was slower than that of the vector group and ALKBH5-R317K group (Fig. 4D), which was consistent with the noticeably lower tumor weights (Fig. 4E).

Histological examination revealed that tumor cells expressing Alkbh5-WT had greater levels of methylated ALKBH5 than other groups (Fig. 4F and G). The location of the tumor in situ and the ability of the tumor cells to proliferate were determined by Ki-67 expression. Noticeably, the immunohistochemistry (IHC) data revealed that, in line with the vector group, the Alkbh5-WT group had fewer Ki-67-positive cells, while the Alkbh5-R317K group had more (Fig. 4F and G). TIIC-related markers, including CD3, CD8a, and GZMB, represent T-cell killing of tumor cells. Strikingly, only mice injected with Alkbh5-WT cells showed higher levels of CD3, CD8a, and GZMB than those in the vector and Alkbh5-R317K groups (Fig. 4F and G). All these findings point to a critical function for meR316-ALKBH5 in regulating the immune evasion of CRC cells both in vivo and in vitro.

### PRMT5-mediated meR316-ALKBH5 activates CD276 transcription by increasing its mRNA m6A modification

**Fig. 5.**
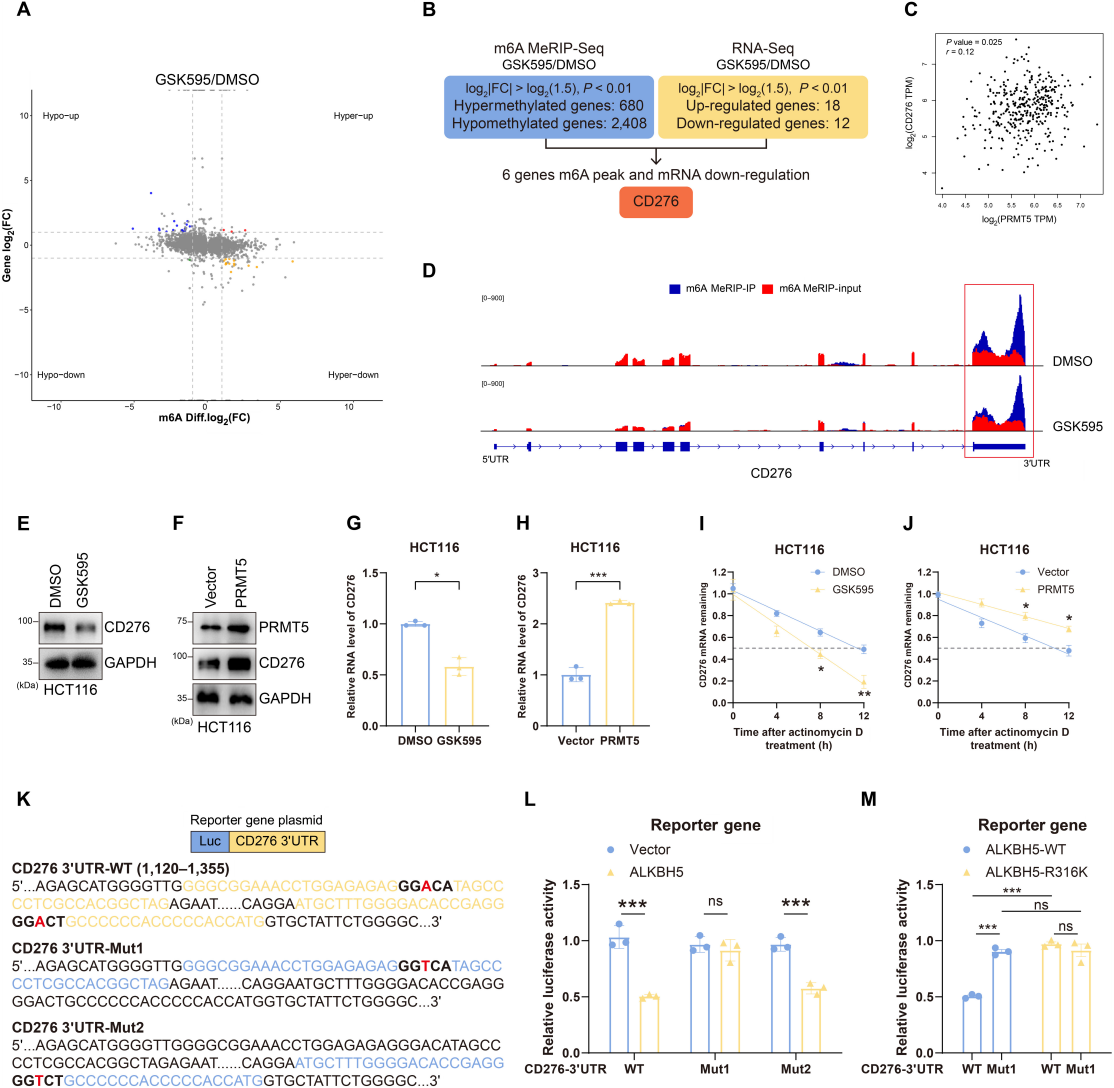
PRMT5 and ALKBH5 regulate CD276 expression in an N6-methyladenosine (m6A)-dependent manner. (A) Distribution of genes with a significant change in both the m6A level and the gene expression level in HCT116 cells treated with GSK595 compared with that in control cells. (B) Schematic of downstream analysis that was mediated by the PRMT5 inhibitor GSK595 in colorectal cancer cells using methylated RNA immunoprecipitation sequencing (MeRIP-seq) and RIP-seq. (C) The expression of PRMT5 is positively correlated with CD276 in colorectal cancer cells verified by the TCGA database. (D) Distribution of m6A peaks across the CD276 mRNA transcript. The 3′ untranslated region (3′UTR) of the CD276 mRNA is highlighted in the red box. (E and F) CD276 protein levels were analyzed by Western blot in HCT116 cells treated by the PRMT5 inhibitor GSK595 or overexpressing PRMT5. (G and H) CD276 mRNA expression were assessed by qRT-PCR assays after HCT116 cells were treated by the PRMT5 inhibitor GSK595 or overexpressed PRMT5. (I and J) The effect of GSK595 or PRMT5 on the stability of CD276 mRNA in HCT116 cells. (K) Schematic diagram of the mutation of the m6A site in CD276 3′UTR. For the CD276 3′UTR-Mut reporter, A–T substitutions (shown in red) were made within m6A consensus. (L and M) Relative luciferase activity of CD276 3′UTR-WT/CD276 3′UTR-Mut reporter when cotransfected with ALKBH5-WT/ALKBH5-R316K expression vector respectively. *P < 0.05; **P < 0.01; ***P < 0.001. FC, fold change; Luc, luciferase.

Since ALKBH5 has been reported to act as an eraser of m6A, the most common modification found in eukaryotic RNAs, and to affect the stability of m6A-modified transcripts [36,37], we sought to identify its downstream effectors mediated by the PRMT5 inhibitor GSK3326595 in CRC cells using MeRIP (m6A) sequencing and RIP sequencing (Fig. 5A). Methylated RNA immunoprecipitation sequencing (MeRIP-seq) revealed 3,088 peaks (680 hypermethylated and 2,408 hypomethylated; fold change = 1.5, *P* < 0.05) in CRC cells (Fig. 5B, blue panel). Next, we used RNA sequencing (RNA-seq) analysis to identify the target gene of PRMT5-mediated ALKBH5, and 30 genes (18 up-regulated and 12 down-regulated; fold change = 1.5, *P* < 0.05) were differentially expressed in HCT116 cells treated with GSK595 (Fig. 5B, yellow panel). Finally, we identified 6 overlapping genes by comparing the MeRIP-seq and RNA-seq results; these genes were associated with m6A modification and down-regulated mRNAs. Among the 6 genes, only CD276 was found to have a significant positive correlation with the expression of PRMT5 (Fig. 5C and Fig. S4A).

The accumulation of m6A modifications throughout the CD276 transcript is depicted in Fig. 5D. Notably, the 3′ untranslated region (3′UTR) of CD276 mRNA exhibited several m6A modifications, which drastically decreased in HCT116 cells treated with GSK3326595 (Fig. 5D). GSK3326595 and PRMT5 governed CD276 at the protein and mRNA levels (Fig. 5E to H). We next investigated whether PRMT5 had an impact on CD276 mRNA stability. HCT116 cells were then treated with actinomycin D, an inhibitor of RNA synthesis. A substantial decrease in the abundance of CD276 mRNA was detected in HCT116 cells treated with GSK3326595 (Fig. 5I). However, CD276 mRNA stability was obviously increased upon PRMT5 overexpression (Fig. 5J).

Because the m6A site of CD276 mRNA was found to be concentrated in the 3′UTR of CD276 mRNA, we used SRAMP to predict 2 methylation sites in the 3′UTR of the CD276 mRNA sequence (Fig. S4B). We next inserted the 3′UTR regions of the CD276 mRNA WT or m6A site mutant (Mut1 and Mut2) sequence into a luciferase reporter (Fig. 5K). We used target–gene reporter luciferase assays with a CD276 mRNA 3′UTR harboring wild-type or mutant m6A sites to determine whether ALKBH5 directly targets CD276 mRNA. When ALKBH5-overexpressing cells were treated with a reporter plasmid containing CD276 3′UTR-WT or CD276 3′UTR-Mut2, luciferase activity decreased; however, CD276 3′UTR-Mut1 did not affect luciferase activity (Fig. 5L).

These findings suggest that the first m6A site is the location of the m6A modification of the CD276 3′UTR. Furthermore, the luciferase activity of the ALKBH5-overexpressing cells treated with CD276 3′UTR-WT dramatically decreased compared with that of the cells treated with CD276 3′UTR-Mut1. However, when we overexpressed ALKBH5-R316K, the difference in luciferase activity between CD276 3′UTR-WT and CD276 3′UTR-Mut1 no longer existed (Fig. 5M). These results suggest that the regulation of CD276 in CRC cells is m6A dependent and is mediated via PRMT5-mediated meR316-ALKBH5.

### PRMT5-mediated ALKBH5-R316 methylation is correlated with poor clinical prognosis in CRC patients

**Fig. 6.**
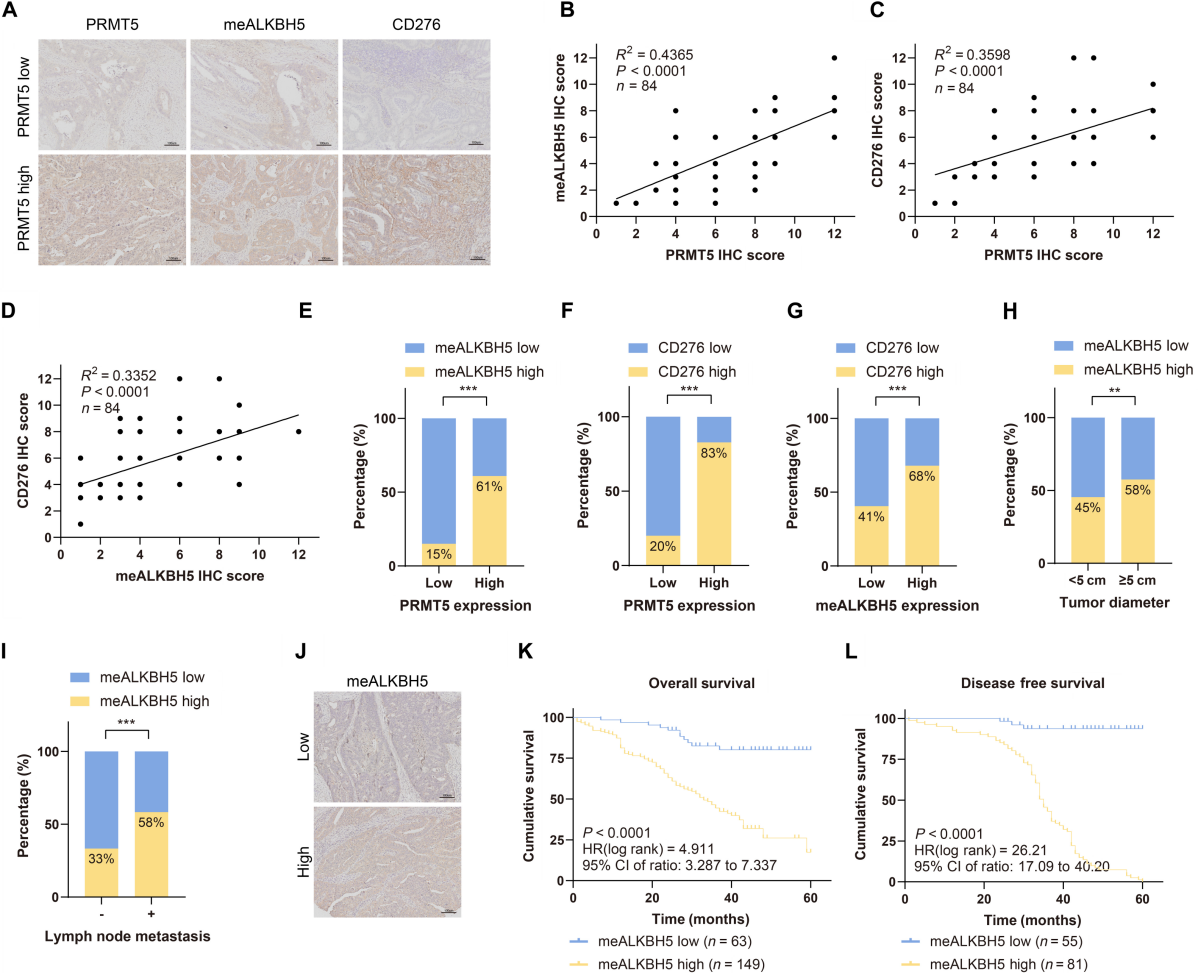
PRMT5-mediated ALKBH5-R316 methylation is associated with poor prognosis in colorectal cancer patients. (A) Representative immunohistochemistry images of PRMT5, meR316-ALKBH5, and CD276 expressions in PRMT5-low case and PRMT5-high case were presented in colorectal cancer patients. (B to D) Correlations between PRMT5 and meR316-ALKBH5 expression (B), PRMT5 and CD276 expression (C), and meR316-ALKBH5 and CD276 expression (D) were examined by Pearson correlation coefficient tests, respectively. (E to G) Correlations between PRMT5 and meR316-ALKBH5 expression (E), PRMT5 and CD276 expression (F), and meR316-ALKBH5 and CD276 expression (G) were examined by Fisher’s exact test, respectively. (H and I) Percentages of a high level of meR316-ALKBH5 expression correlated with tumor sizes (H) and lymph node metastasis (I) as examined by *χ*^2^ tests. (J) Representative images of weak and strong meR316-ALKBH5 staining in colorectal cancer tissues. (K) High meR316-ALKBH5 expression correlated with a poorer 5-year overall survival for 212 colorectal cancer patients (*P* < 0.0001, log-rank test). (L) High meR316-ALKBH5 expression correlated with a poorer 5-year disease-free survival for 136 colorectal cancer patients (*P* < 0.0001, log-rank test). **P < 0.01; ***P < 0.001. CI, confidence interval; HR, hazard ratio.

We conducted IHC analyses of CRC tissue microarrays (84 samples) to detect the PRMT5, meR316-ALKBH5, and CD276 expression levels and further examined their relevance and clinical significance. We revealed that between PRMT5 and meR316-ALKBH5, the PRMT5 and CD276 levels and the meR316-ALKBH5 and CD276 levels were positively correlated (Fig. 6A to D). Furthermore, we found that high PRMT5 expression was strongly correlated with meR316-ALKBH5 and CD276 expression (Fig. 6E and F). Finally, we also found that there was a positive correlation between high CD276 expression and high meR316-ALKBH5 expression (Fig. 6G). These results supported our hypothesis that by increasing CD276 expression, PRMT5-mediated ALKBH5-R316 methylation promotes the progression of CRC.

To further verify the above results, meR316-ALKBH5 expression was examined in an expanded CRC cohort (tissue microarray, 212 samples). Using IHC assays, we evaluated the associations between meR316-ALKBH5 levels and clinicopathological features. First, the data demonstrated a strong correlation between tumor size and lymph node metastasis and high levels of meR316-ALKBH5 (Fig. 6H and I). The associations between meR316-ALKBH5 expression and other related clinicopathological features were investigated using Pearson’s chi-square test. High meR316-ALKBH5 expression was significantly positively correlated with TNM stage (*P* < 0.0001), depth of invasion (*P* < 0.0001), and metastasis (*P* = 0.008) (Table S4). Specifically, meR316-ALKBH5 expression, tumor diameter, differentiation, TNM stage, depth of invasion, lymph node metastasis, and metastasis were found to be important predictive variables for the overall survival (OS) and disease-free survival (DFS) of patients with CRC according to univariate Cox regression analysis (Table S5).

Moreover, meR316-ALKBH5 expression remained an independent predictor of OS and DFS in CRC patients according to the multivariate Cox regression model (Table S6). Finally, we showed that high levels of meR316-ALKBH5 were strongly correlated with poor 5-year OS and DFS (Fig. 6J to L). These findings demonstrate a substantial correlation between meR316-ALKBH5 and the clinical aggressiveness of CRC, hence supporting the function of meR316-ALKBH5 in the clinical behavior of human CRC.

### The PRMT5 inhibitor GSK3326595 combined with an anti-PD1 antibody dramatically prevents CRC immune evasion

**Fig. 7.**
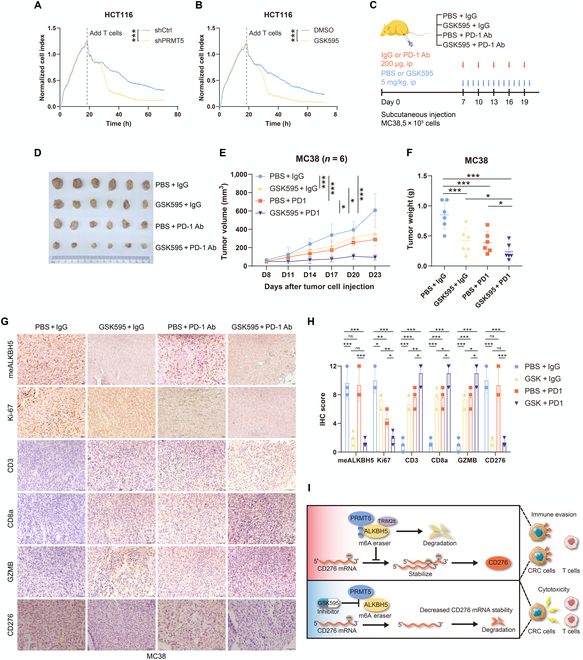
GSK3326595 treatment eradicates colorectal cancer cells’ progress when anti-PD-1 is concurrently applied. (A) Down-regulated PRMT5 in HCT116 cells evaluates the specific cytotoxicity of T cells. (B) PRMT5 inhibitor GSK595 promotes the cytotoxicity of T cells targeting colorectal cancer cells. (C) Schematic diagram of C57BL mouse implanted with MC38 cells under different treatments. (D and F) Representative images (D), tumor growth curves (E), and tumor volume (F) of different treatments in each group. (G) Representative images of the IHC staining of meALKBH5, Ki-67, CD3, CD8a, GZMB, and CD276 in the tumor xenografts. (H) Quantification of meALKBH5, Ki-67, CD3, CD8a, GZMB, and CD276 staining intensity in xenograft tumors. (I) Proposed model to describe the role of PRMT5-mediated ALKBH5-R316 methylation in promoting m6A-dependent CD276 activation.*P < 0.05; **P < 0.01; ***P < 0.001. Ab, antibody; ip, intraperitoneal; PBS, phosphate-buffered saline.

Recently, PRMT5 has become an intriguing treatment option for treating cancer. GSK3326595, a PRMT5 inhibitor, is being tested in clinical trials to treat various malignancies. We next examined whether pharmacological or genetic inhibition of PRMT5 could mediate specific T-cell cytotoxicity to CRC cells. Notably, treatment with shPRMT5 or GSK3326595 dramatically reduced HCT116 cell survival in a T-cell killing assay in vitro (Fig. 7A and B).

The possibility of synergistic suppression of CRC advancement by both anti-PD-1 therapy and PRMT5 inhibition was investigated. A xenograft mouse model was generated in C57BL mice (Fig. 7C). Notably, compared to the combination of GSK3326595 or anti-PD-1 alone, the combination of GSK3326595 and anti-PD-1 significantly decreased the rate of CRC growth (Fig. 7D to F). Importantly, our IHC data showed that the combination of GSK3326595 and PD-1 blockade cooperatively decreased the Ki-67 and CD276 levels in MC38 tumor cells and increased the levels of TIIC-related markers, including CD3, CD8a, and GZMB (Fig. 7G and H), which indicate T-cell infiltration while preventing cytotoxicity, leading to improved tumor rejection. These findings indicate that PRMT5 inhibits the full response of CRC to anti-PD-1 treatment, highlighting the importance of the combination of GSK3326595 and PD-1 blockade in immunotherapy.

## Discussion

The methylation of arginine in proteins is crucial for the development of many types of cancer. Drug resistance and tumor heterogeneity are increased by dysregulated protein arginine methyltransferase activity. From this perspective, it is imperative that we can obtain a thorough understanding of the molecular mechanism underlying CRC [38]. Accumulating evidence indicates that protein arginine methylation can either positively or negatively affect protein and RNA interactions to regulate biological functions [39]. The expression of PRMT5, a critical epigenetic regulator of PTMs, is elevated in several cancers, particularly in CRC, and this increased expression is linked to a low patient survival rate [40].

In this study, we first outline the vital function of PRMT5-mediated R316-ALKBH5 SDMA in ALKBH5 protein degradation and its ability to increase CD276 mRNA stability during CRC immune evasion. We found that PRMT5 depletion (both in the context of GSK3326595 treatment and PRMT5 knockdown) reduces the m6A level in CRC cells. We discovered that ALKBH5 is a crucial substrate for methylation mediated by PRMT5, leading to its binding with TRIM28 and its ubiquitination-mediated degradation. We proved that in CRC cells, PRMT5-mediated meR316-ALKBH5 enhanced m6A modification on the 3′UTR of CD276 mRNA, thereby increasing the CD276 protein level. We illustrated the restriction of CRC immune evasion by meR316-ALKBH5. The complex role of R316-ALKBH5 in the CRC tumor immune microenvironment was revealed. We investigated the potential positive correlations between high levels of meR316-ALKBH5 and TNM stage, depth of invasion, lymph node metastasis, and tumor size in CRC patients. We showed a striking correlation between low 5-year OS and DFS and high levels of meR316-ALKBH5. These findings demonstrate a direct correlation between meR316-ALKBH5 and the clinical aggressiveness of CRC and reaffirm the function of meR316-ALKBH5 in the clinical outcome of CRC.

Interestingly, a report showed that ALKBH5 is directly methylated at the R283 site by the ADMA modification of PRMT6, which thus promotes the formation of breast tumors [41]. However, we found that ALKBH5 is distinctly methylated by PRMT5 via SDMA methylation at the R316 site in CRC cells. Taken together, these findings indicate that R283 ADMA or R316 SDMA of ALKBH5 can also lead to tumor development, which means that arginine methylation of ALKBH5 is vital for tumorigenesis. The function of ALKBH5 in cancer is complicated and debatable; reports suggest that it may have either tumor-suppressive or carcinogenic properties [42]. ALKBH5 caused an increase in suppressive immune cells and lactate levels, which increased the susceptibility of tumors to anti-PD-1 therapy [43]. CD276 is overexpressed in cancer cells, inhibits T-cell activation, and has nonimmune functions [44–46].

Clinical investigations conducted most recently have demonstrated that GSK3326595 has exceptional anticancer properties and works against several types of carcinomas (NCT02783300 and NCT03614728). These findings indicate that GSK3326595 is a promising affordable therapeutic option for CRC patients. As anti-PD-1 monotherapy has limited clinical activity in CRC patients, our study showed that the combination of GSK3326595 and anti-PD-1 restrains CRC cell progression and provides a rational alternative solution for CRC patients to achieve better clinical outcomes.

It is intriguing that ALKBH5 is a primary m6A demethylase, which is dysregulated and has a biological and pharmacological role in human cancers. ALKBH5 plays a dual role in various cancers, impacting positive and negative substrates by regulating kinds of biological processes. The underlying mechanisms of ALKBH5 in human cancers were not only unclear but also controversial. Up to now, the expression of ALKBH5 has been found to be up-regulated or down-regulated in various cancers and to play an oncogenic or tumor suppressive role in CRC and so on. All evidence suggests that the role of ALKBH5 in the prognosis of cancers and biofunction might be context dependent.

In summary, we are the first to report that the interactions between PRMT5, meR316-ALKBH5, and TRIM28 strongly affect ALKBH5 protein stability via PTMs. Through a series of functional studies conducted both in vivo and in vitro, we demonstrated in our study that the PRMT5–meR316-ALKBH5–CD276 axis promotes CRC carcinogenesis and revealed that meR316-ALKBH5 expression in CRC affects tumor immune evasion. In addition, we assessed the potential therapeutic value of combining GSK3326595 and anti-PD1 to effectively suppress the progression of CRC and enhance treatment outcomes in preclinical tumor models. This study’s findings highlight the role of R316-ALKBH5 methylation in CRC immune evasion and point to the potential utility of meR316-ALKBH5 as a biomarker for CRC diagnosis and treatment. However, more research needs to be done to determine how altering epigenetic regulators affects tumor immunotherapy. Further studies with multiple CRC cell lines and mouse models are needed to prove the efficacy of PRMT5 inhibitors.

## Materials and Methods

### Cell culture and treatment

All cell lines were acquired from the Chinese Academy of Sciences Cell Library. The cell treatments used are detailed in the Supplementary Materials and Methods.

### Western blot, IP, and Co-IP analyses

Western blot, IP, and Co-IP analyses were performed as previously described [18]. The Supplementary Materials and Methods section contains a list of the methods and antibody details.

### m6A dot blot

A total of 200 ng or 400 ng of mRNA obtained by dilution was denatured, and the secondary structure was disrupted at 95 °C for 3 min. The mixture was immediately cooled to prevent the secondary structure from reforming. The mRNA was transferred onto a nylon membrane, which was then gently washed with DEPC water. The spotted mRNA was cross-linked with the membrane twice using an ultraviolet-linked immunosorbent assay. The membrane was incubated for 1 h at room temperature in 10 ml of blocking buffer. Then, the membrane was incubated with 10 ml of m6A antibody dilution buffer overnight at 4 °C. The membrane was washed 3 times with 10 ml of detergent for 5 min. The anti-rabbit IgG–HRP membrane was incubated gently at room temperature at a dilution of 1:5,000 for 1 h. After that, the membrane was incubated with 3 ml of Hyperfilm ECL reagent at room temperature in the dark for 5 min before exposure.

### MeRIP-seq and RNA-seq

To perform RNA-seq and MeRIP-seq, total RNA from HCT116 cells treated with GSK3326595 and control cells was extracted using TRIzol reagent and submitted to LC-Bio. The Supplementary Materials and Methods provide information on the MeRIP-seq and RNA-seq analysis.

### Measurement of the m6A/A ratio by LC–MS/MS

The nucleoside samples were then subjected to liquid chromatography coupled with tandem mass spectrometry (LC–MS/MS). By comparing the standard curves derived from the respective nucleoside standards, the concentrations of m6A and A were ascertained. The computed concentrations were used to assess the m6A-to-A ratio. The Supplementary Materials and Methods provide a full description of the method.

### GST pull-down assay and in vitro methylation assay

Following a previously published protocol [18], GST, GST–ALKBH5, and GST–ALKBH5 (292 to 395 AA) fragmented proteins were expressed in bacteria (BL21) stimulated with isopropyl-β-d-thio-galactoside and then purified. The Supplementary Materials and Methods contain detailed protocols for the in vitro methylation assay.

### Immunohistochemistry

IHC was carried out using the conventional streptavidin–peroxidase protocol, as previously described [18]. The Supplementary Materials and Methods include a full description of the antibody and IHC assessment methods.

### Animal works

The animal experiments were approved by the Animal Care and Use Committee of Xuzhou Medical University, China. We purchased female C57BL mice aged 6 to 8 weeks from Beijing Vital River Laboratory Animal Technology Co., Ltd. The Supplementary Materials and Methods provide a full description of the assessment.

### Clinical tissue specimens

The Supplementary Materials and Methods provide information about the CRC patient specimens.

### Statistical analysis

Statistical analysis was performed using GraphPad Prism 9.0. As specified, error bars display the mean ± standard error of the mean or mean ± SD. Unless otherwise stated, a Student 2-tailed unpaired *t* test with 95% confidence intervals was used to assess significance. *P < 0.05; **P < 0.01; ***P < 0.001; ns, not significant.

## Data Availability

The data supporting the findings of this study are available from the corresponding authors upon reasonable request.
