# Supplementary material for: PRMT5-Mediated ALKBH5 Methylation Promotes Colorectal Cancer Immune Evasion via Increasing CD276 Expression
Source: Research (Wash D C). 2025 Jan 8;8:0549. doi: 10.34133/research.0549 (PMC11707101; doi:10.34133/research.0549)
Supplement: Supplementary 1 — Supplementary Materials and Methods Figs. S1 to S4 Tables S1 to S6 [file research.0549.f1.zip › Supplementary Materials and Methods.docx]

**PRMT5-mediated ALKBH5 methylation promotes colorectal cancer immune evasion via increasing CD276 expression**

Sen Meng^1,2#^, Hao Liu^2#^, Jiayu Xu^5#^, Chuyin Deng^1^, Xingyou Qian^1^, Sufang Chu^1^, Wei-Guo Zhu^4,6^, Jiuling Zhu^4^, Hongmei Yong^5^*, Zhongwei Li^4,7^* and Jin Bai^1,2,3^*

^1^Cancer Institute, Xuzhou Medical University, Xuzhou, Jiangsu, China.

^2^Centre of Clinical Oncology, the Affiliated Hospital of Xuzhou Medical University, Xuzhou, Jiangsu, China.

^3^Jiangsu Center for the Collaboration and Innovation of Cancer Biotherapy, Cancer Institute, Xuzhou Medical University, Xuzhou, Jiangsu, China.

^4^Laboratory of Epigenetic Regulation in Molecular Medicine, Department of Pathophysiology, School of Basic Medical Sciences, Wannan Medical College, Wuhu, Anhui, China.

^5^Department of Oncology, The Affiliated Huai'an Hospital of Xuzhou Medical University and The Second People's Hospital of Huai'an, Huai’an, Jiangsu, China.

^6^International Cancer Center, Guangdong Key Laboratory of Genome Instability and Human Disease Prevention, Marshall Laboratory of Biomedical Engineering, Department of Biochemistry and Molecular Biology, Shenzhen University Medical School, Shenzhen, China.

^7^Anhui Province Key Laboratory of Basic Research and Transformation of Age-related Diseases, Wannan Medical College, Wuhu, Anhui, China.

**Authorship notes:** #These authors contributed equally to this work.

**Supplementary Materials and Methods**

**Cell culture and treatment**

10% FBS was added to the RPMI-1640 media when cultivating the DLD1 cells. MC38, HCT116, and HEK293T cells were grown in DMEM supplemented with 10% FBS.

The small interfering RNAs (siRNA, 50 nM) against human PRMT5 were transfected into the CRC cells with SilenFect reagent (Thermo Fisher Scientific Inc., USA), while non-specific siRNA was used as negative controls. All siRNAs were purchased from Gene pharma Technology (Shanghai, China).

T cells isolated from healthy human peripheral blood mononuclear cells (PBMC). Isolated T cells were then cultured in RPMI-1640 medium supplied with human CD3/CD28 magnet beads (11161D, Thermo Fisher Scientific) and 30U/mL recombinant human IL-2 (589104, BioLegend) for expansion and activation for 3 days.

**Lentiviral production and infection**

Lentiviruses were produced by co-transfecting HEK293T cells with one of the expression plasmids and the packaging plasmids (psPAX2 and pMD2.G). The supernatants were collected after 48 hours, and filtered through 0.45μm filters (Millipore, Temecula, CA, USA), then concentrated using Amicon Ultra centrifugal filters (Millipore 100KD MWCO). The concentrated viruses were used to infect HCT116, DLD1 and MC38 cells. Stable transfection cell lines were selected with 1mg/ml puromycin for 15 days.

**Western blot**

Total protein from cells was extracted by using RIPA lysis buffer and was qualified by using a BCA detecting kit (Keygen, Nanjing, China). Proteins samples were subjected to 10% SDS-PAGE and transferred onto a NC membrane, and then incubated with specific antibody at 4℃ overnight, respectively. The next day, the membranes were incubated with secondary antibodies included HRP-goat anti-mouse, HRP-goat anti-rabbit (ABclonal) at room temperature for 1h. Protein bands were detected on Tanon 5200. Automatic chemiluminescence imaging analysis system using ECL reagent (Tanon, Shanghai, China).

**Immunoprecipitation and co-immunoprecipitation**

Cell lysates were obtained by incubating the cells in lysis bufer (50mM Tris–HCl, pH8.0; 0.2% NP-40, 150mM; NaCl, 2mM, EDTA and protease inhibitor cocktail) for 20min at 4℃, followed by centrifugation at 14,000 g for 15min at 4℃. Overall, 5% whole-cell extracts were used for input. The rest of the protein extracts were incubated with 2μg control or specific antibodies overnight at 4℃. Then, 10μL of Anti-Myc Nanobody Agarose beads / Anti-Flag Nanobody Agarose beads (KT HEALTH KTSM1306/KTSM1308) were added with further incubation at 4℃ for 2h. Beads were then washed five times using the cold lysis buffer. The immunoprecipitates were boiled with 2×SDS-PAGE loading bufer, separated on SDS-PAGE gels, followed by immunoblotting with various antibodies indicated.

**Antibodies and reagents**

The specific antibodies and reagents used in this study were as follows: Antibody against GAPDH was used as control (Proteintech, Cat #60004-1-Ig). PRMT5 (Proteintech, Cat #18436-1-AP), ALKBH5 (Proteintech, Cat #67811-1-Ig), TRIM28 (Proteintech, Cat #66630-1-Ig), CD276 (Cell Signaling Technology, Cat #14058), SDMA (Cell Signaling Technology, Cat#13222), MMA (Cell Signaling Technology, Cat#8015),Ub (Cell Signaling Technology, Cat#3933)were used for Western blot assays. The anti-SDMA-R316-ALKBH5 (anti-meR316-ALKBH5) antibody was raised against the region near R316 symmetric dimethylarginine site of ALKBH5. The symmetric di-methylated synthetic peptide [LSGNNR(R-Me2)DPALKPK-C] was used for immunization in the rabbit. The antibody was generated in Gl biochem company (Shanghai, China). GSK3326595 (Selleck, S8664) were purchased from Selleck (Houston, Texas, USA).

**RNA extraction and quantitative real-time PCR (qRT-PCR) assay**

Total RNA from cell and tissue samples was isolated using TRIzol Reagent (Invitrogen, USA) according to the manufacturer’s protocols, and cDNA was synthesized using the HiScript 1st Strand cDNA Synthesis Kit (Vazyme Biotech, Nanjing, China). Quantitative realtime PCR was carried out on ABI-7500 using UltraSYBR One Step RT-qPCR Kit (CWBIO, Beijing, China). The relative mRNA expression levels were normalized to and GAPDH. The primers are listed in Supplemental Materials.

**Identification of ALKBH5 methylation sites by mass spectrometry**

To identify in vivo methylation sites of ALKBH5, HEK293T cells were co-transfected with Flag-ALKBH5 and PRMT5. The cell lysates were coimmunoprecipitated with an anti-Flag antibody. The immunoprecipitated Flag-ALKBH5 was subjected to SDS-PAGE. And the bands corresponding to ALKBH5 were subjected to in-gel trypsin digestion. The labeled peptides were analyzed with Liquid chromatography-tandem mass spectrometry analysis (LC-MS/MS) performed in the APPLIED PROTEIN TECHNOLOGY, Shanghai, China.

**MeRIP-seq and RNA-seq**

For RNA-seq, total RNA from HCT116 cells treated with GSK3326595 and corresponding control cells were extracted using TRizol Reagent and submitted to the LC-Bio for RNA-sequencing and Methylated RNA immunoprecipitation sequencing (MeRIP-seq). Briefly, mRNA was purified using the NEBNext Poly(A) mRNA Magnetic Isolation Kit (E7490S, NEB). RNA fragmentation was performed by incubation with magnesiumions at 94℃ using NEBNext Magnesium RNA Fragmentation Module (E6150S, NEB). The specific anti-m6A antibody was applied for m6A pull down. The enriched mRNA fragments were then used to construct libraries with VAHTS Total RNA-seq (H/M/R) Library Prep Kit for Illumina (NR603, Vazyme). Sequencing was carried out on an Illumina HiSeq 2500 with paired-end 150-bp read length.

**Measurement of the m6A/A ratio by LC-MS/MS**

RNA sample were digested into single nucleosides in a digestion buffer containing phosphodiesterase I (0.01 U), nuclease S1 (180 U), 1mM zinc sulfate, 280mM sodium chloride and 30mM sodium acetate at pH6.8 for 4h at 37℃, and dephosphorylated with bacterial alkaline phosphatase (30 U) for 2h at 37℃. Enzymes were removed by filtration (Amicon Ultra 10K MWCO). The nucleosides samples were then subjected to liquid chromatography coupled with tandem mass spectrometry (LC-MS/MS) The m6A and A concentrations were determined by comparison with the standard curves obtained from their nucleoside standards. The ratio of m6A to A was analyzed based on the calculated concentrations.

**GST pull‑down assay**

GST fusion proteins were transformed in *E. coli* (BL21) and induced with 1mM IPTG at 37℃ for 3h. The cells were harvested by centrifugation at 4,000 rpm for 10 min. Then, the bacterial pellets were resuspended and lysed by sonication in cold PBS in the presence of complete protease inhibitor cocktail, followed by centrifugation to collect the supernatant at 12,000g for 10 min at 4℃. The cell lysates were applied to prepared glutathione agarose beads (MCE Cat#HY-K0211) and incubated for 2h at 4℃. Beads were washed five times using the cold PBS. Glutathione agarose beads GST fusion proteins were mixed with Myc-PRMT5 proteins, which were purified from HEK293T cells, and incubated for 2h at 4℃. The beads were then washed three times with wash buffer (50 mM Tris–HCl, pH 8.0, 0.2% NP-40, 150 mM NaCl, 2 mM EDTA). The bound proteins were eluted by boiling in 2×SDS-PAGE loading buffer, followed by immunoblotting analysis. The purified GST fusion proteins were examined for the presence by coomassie brilliant blue staining.

**In vitro methylation assay**

In vitro methylation assay conditions: 0.5 ug Histone H4 (Cat. No. 31493, Proteintech, USA) was incubated with 1ug of PRMT5/MEP50 (Cat No: 31521,31921, Proteintech, USA) Complex in 30ul reaction system including 50 mM Tris-HCl pH 8.6, 0.02% Triton X-100, 2 mM MgCI_2_, 1 mM TCEP, and 50 uM SAM for 3h at room temperature. GST-ALKBH5 (residues 292-395AA) as a substrate was incubated with the PRMT5/MEP50 Complex in mentioned condition. The reaction was stopped with SDS loading buffer and activity was detected by Western Blot.

**T-cell killing assay in vitro and Real time cellular analysis (RTCA)**

The activated T-cell were cocultured with tumor cells. After incubation, the viability of tumor cells was measured by Real time cellular analysis (RTCA xCelligence, USA). The xCELLigence system was used according to the instructions of the supplier (ACEA Biosciences). The system measured impedance differences to derive cell index values at time points and it may be set by the operator. The xCELLigence system consists of four main components: the RTCA analyzer, the RTCA DP station, the RTCA computer with integrated software and disposable E-plate 16. Firstly, the optimal seeding concentration for proliferation assay was determined. After seeding the number of 5000 cells in 200 ml medium to each well in E-plate 16, the attachment and proliferation of the cells were monitored every 15min. All experiments were carried out for 96h.

**Immunohistochemistry (IHC)**

In IHC assays, heat-induced epitope retrieval was performed with retrieval buffer (EDTA, pH9.0 or citrate, pH6.0). The primary antibodies used in IHC are described as follows: meR316-ALKBH5 antibody, Ki-67 antibody (ab51608, abcam), CD3 antibody (17617-1-AP, Proteintech), CD8a antibody (A23081, ABclonal), GZMB antibody (13588-1-AP, Proteintech). Briefly, the detailed IHC assessment method of TMAs was performed as the staining scores of meR316-ALKBH5 were evaluated via combining the percentage of cells with the staining intensity and the IRS (immunoreactivity score, IRS) by three pathologists separately. The intensity of meR316-ALKBH5 immunostaining was scored as 0–3 (0, negative; 1, weak; 2, moderate; 3, strong); the percentage of immunoreactivity cells was graded as 1 (0–25%), 2 (26–50%), 3 (51–75%), and 4 (76–100%). Relied on the IRS, the level of meR316-ALKBH5 expression was categorized as low (IRS: 0–4) and high (IRS: 6–12) expression.

**RNA stability**

At different times, 10 µg/mL actinomycin D (Millipore, Billerica, MA, USA), a transcriptional inhibitor, was added to the cells for incubation. Next, the relative abundance of each mRNA was measured by qRT‒PCR.

**Dual-luciferase reporter assay**

The specified reporter plasmid was transfected into cells that had been plated in a 48-well plate. Following the manufacturer's instructions, the Dual-Luciferase Reporter Assay System (Promega, catalogue no. E1910) was used to measure the activity of firefly luciferase and Renilla luciferase in each well. Renilla luciferase and firefly luciferase activities were measured. The ratio of firefly luciferase activity to Renilla luciferase activity was considered the relative luciferase activity.

**Bioinformatics prediction and analysis**

We investigated the relationship between immune infiltrates and ALKBH5 in the TCGA cohort using TIMER 2.0 (http://timer.comp-genomics.org/timer/). The Cancer Genome Atlas (TCGA) and Gene Expression Profiling Interactive Analysis (GEPIA) (http://gepia.cancer-pku.cn/) databases were utilized to examine the relationship between PRMT5 and CD276 in CRC. We obtained CD276 transcript sequences from the NCBI website (https://www.ncbi.nlm.nih.gov/), and we used SRAMP (http://www.cuilab.cn/sramp) to predict the local structures and m6A modification sites.

**Animal Works**

We used C57BL mice (Vital River Laboratory Animal Technology. China) for research. We subcutaneously injected MC38 cells (5×10^6^) of each group into the two side of mice. When tumors were visible, we recorded xenograft tumors volume (V) every 3 days by metering the long axis (L) and the short axis (W), and calculated the tumors growth rate with the equation: V = (L × W^2^)/2. The xenograft tumor tissues were harvested, weighed, photographed and subjected to subsequent analysis. Animal Care and Use Committee and Ethics Committee of Xuzhou Medical University approved all animal experiments.

**Clinical tissue specimens**

This study was approved by the Ethical Committee of the Affiliated Hospital of Xuzhou Medical University from 2010 to 2015 in China. Written consent was obtained prior to subject enrollment, following informed consent at the time of acquisition. Clinical and pathological information was obtained from the medical records of the Affiliated Hospital of Xuzhou Medical University. Survival time was calculated based on the date of surgery to the date of death or to the last follow-up. The patient studies were conducted in accordance with Declaration of Helsinki. The use of these specimens and data for research purposes were granted approval by the Ethics Committee of the Affiliated Hospital of Xuzhou Medical University.

**Ethics approval and consent to participate**

This study was conducted in compliance with the principles of the Declaration of Helsinki. Informed consent was obtained from all the subjects. Ethics approval for human subjects was provided by the Ethics Committee of the Affiliated Hospital of Xuzhou Medical University. Ethics approval for animal work was provided by the Institutional Animal Care and Use Committee of Xuzhou Medical University.

**Consent for publication**

Not applicable.

**Supplementary Figure legend**

**Supplementary Figure 1 PRMT5 regulate m6A global level in** **colorectal cancer cells**

**(A)** The ratio of modified nucleosides to corresponding nucleosides in comparison group of GSK595/DMSO in colorectal cancer cells, log2(ratio) was taken to symmetrize the ratio, log2(ratio) >0 means that nucleoside is upregulated in the comparison group, and log2(ratio) <0 means that nucleoside is downregulated in the comparison group. The abatement of m6A was most obvious.

**(B)** Quantitative analysis shown that the value of m6A/A was lower in GSK595 than DMSO of colorectal cancer cells. **P* < 0.05, ***P* < 0.01, ****P* < 0.001.

**Supplementary Figure 2 PRMT5 weakens the stability of ALKBH5 by increasing its ubiquitination**

**(A)** Immunoafnity purifcation and mass spectrometry analysis of PRMT5-binding proteins. The eluates were resolved by SDS-PAGE and visualized by coomassie staining. The protein bands on the gel were excised and identifed by mass spectrometry.

**(B)** The purifed fractions were analysed by western blotting with antibodies against indicated proteins.

**(C)** The protein expression of genes involved in m6A modification were analyzed when GSK595 was applied to HCT116, DLD1 and MC38 cells.

**(D-E)** The effect of PRMT5 pharmacological (GSK595) or genetic (PRMT5 konckdown) inhibition on ALKBH5 protein expression was detected by Western-blot in MC38 cells following treated by CHX (50μg/mL) for the indicated time (Left). The relative intensity of ALKBH5 proteins were quantified by software Image J (Right). For normalization, GAPDH expression was used as a control.

**Supplementary Figure 3 meR316-ALKBH5 strengthens E3 ligase TRIM28-mediated ALKBH5 ubiquitination degradation**

**(A)** SDMA and MMA of endogenous ALKBH5 in MC38 cells were assessed by IP assays.

**(B)** MS analysis of ALKBH5 methylation. The fragmentation of the ALKBH5 peptide identified with a dimethylated arginine residue (left table). Schematic illustration showing MS analysis of ALKBH5 methylation. The fragmentation of the ALKBH5 peptide identified with a methylated arginine residue (right panel).

**(C-D)** Elisa and Dot blots result for Antigen Affinity purified Pab (antibody) for anti-meR316-ALKBH5.

**(E)** The ALKBH5 expression was detected by western blotting after expression of Alkbh5-WT or Alkbh5-R317K in MC38 cells treated with CHX (50μg/ml). The bands of ALKBH5 proteins treated by CHX were quantified by software Image J.

**(F)** Western blots of Flag-Alkbh5-associated ubiquitination after IP Alkbh5-Ub in MC38-Alkbh5-WT cells and MC38-Alkbh5-R317K cells. **P* < 0.05, ***P* < 0.01, ****P* < 0.001.

**(G-H)** Western blot analysis of endogenous interaction between ALKBH5 and TRIM28, TRIM25, USP14 relatively after IP ALKBH5 in HCT116 and MC38 cells.

**(I)** Western blot analysis of exogenous interaction between ALKBH5 and TRIM28 after IP Tagged-ALKBH5 in MC38 cells.

**(J-K)** Western blot and qPCR were used to detect ALKBH5 protein and mRNA expression when TRIM28 was overexpressed in HCT116 and MC38 cells.

**(L)** Trim28 overexpression decreased ALKBH5 protein half-life. CHX (50μg/ml) was added in MC38 and MC38 overexpressing Trim28 cells at the indicated time points. Cell lysates were then subjected to immunoblotting analyses.

**(M)** Western blot showing effects of the proteasome inhibitor MG132 (10μM/mL for 8h) treatment on ALKBH5 protein accumulation in MC38 cells. **P* < 0.05, ***P* < 0.01, ****P* < 0.001.

**(N)** TRIM28 mediated ubiquitination degradation of ALKBH5 in HCT116 cells by IP.

**(O)** Western blot analysis of ALKBH5 expression in HCT116 cells stably expressing wild-type ALKBH5 or ALKBH5 R316K treated with MG132 (100 µM), leupeptin (20 µM) or MLN4924 (20 µM) for 6 hours as indicated.

**Supplementary Figure 4 PRMT5 and ALKBH5 regulate CD276 expression in colorectal cancer cells**

**(A)** Correlation analysis between DNAH17−AS1, POTEI, SDCBP2−AS1 and PRMT5 expression in colorectal cancer cells.

**(B)** Prediction score distribution along the query sequence of CD276-3′UTR in SRAMP. High confidences are labeled purple.
