# Supplementary material for: PRMT5-Mediated ALKBH5 Methylation Promotes Colorectal Cancer Immune Evasion via Increasing CD276 Expression
Source: Research (Wash D C). 2025 Jan 8;8:0549. doi: 10.34133/research.0549 (PMC11707101; doi:10.34133/research.0549)
Supplement: Supplementary 1 — Supplementary Materials and Methods Figs. S1 to S4 Tables S1 to S6 [file research.0549.f1.zip › Supplementary Table 1.docx]

**Supplementary Table 1** list of nucleosides and modified nucleosides

| No. | Short name | Full name | CAS | Molecular formula |
| --- | --- | --- | --- | --- |
| 1 | A | Adenosine | 58-61-7 | C10H13N5O4 |
| 2 | U | Uridine | 58-96-8 | C9H12N2O6 |
| 3 | C | Cytidine | 65-46-3 | C9H13N3O5 |
| 4 | G | Guanosine | 118-00-3 | C10H13N5O5 |
| 5 | m6A | 6-Methyladenosine | 1867-73-8 | C11H15N5O4 |
| 6 | m1A | 1-Methyladenosine | 15763-06-1 | C11H15N5O4 |
| 7 | Am | 2'-O-Methyladenosine | 2140-79-6 | C11H15N5O4 |
| 8 | m6Am | 2'-O-Methyl-6-methyladenosine | 57817-83-1 | C12H17N5O4 |
| 9 | m5C | 5-Methylcytidine | 2140-61-6 | C10H15N3O5 |
| 10 | m3C | 3-Methylcytidine | 2140-64-9 | C10H15N3O5 |
| 11 | Cm | 2'-O-Methylcytidine | 70475-20-6 | C10H15N3O5 |
| 12 | hm5C | 5-Hydroxymethylcytidine | 19235-17-7 | C10H15N3O6 |
| 13 | m3U | 3-Methyluridine | 2140-69-4 | C10H14N2O6 |
| 14 | m5U | 5-Methyluridine | 1463-10-1 | C10H14N2O6 |
| 15 | Um | 2'-O-Methyluridine | 2140-76-3 | C10H14N2O6 |
| 16 | m5Um | 2'-O-Methyl-5-methyluridine | 55486-09-4 | C11H16N2O6 |
| 17 | m2G | 2-Methylguanosine | [2140-77-4](https://commonchemistry.cas.org/detail?cas_rn=2140-77-4) | C11H16N5O5 |
| 18 | m7G | 7-Methylguanosine | 20244-86-4 | [C11H15N5O5](https://pubchem.ncbi.nlm.nih.gov/#query=C11H15N5O5) |
| 19 | Gm | 2'-O-Methylguanosine | 2140-71-8 | C11H15N5O5 |
