# Supplementary material for: PRMT5-Mediated ALKBH5 Methylation Promotes Colorectal Cancer Immune Evasion via Increasing CD276 Expression
Source: Research (Wash D C). 2025 Jan 8;8:0549. doi: 10.34133/research.0549 (PMC11707101; doi:10.34133/research.0549)
Supplement: Supplementary 1 — Supplementary Materials and Methods Figs. S1 to S4 Tables S1 to S6 [file research.0549.f1.zip › Supplementary Table 2.docx]

**Supplementary Table 2** Nucleoside quantitation information (content unit: pg)

| Sample | DMSO_1 | DMSO_2 | DMSO_3 | GSK595_1 | GSK595_2 | GSK595_3 |
| --- | --- | --- | --- | --- | --- | --- |
| A | 97568.3 | 91711.2 | 95573.0 | 113960.0 | 94789.2 | 84136.7 |
| U | 156403.4 | 158191.2 | 165205.9 | 197564.3 | 147886.4 | 139351.7 |
| C | 87441.6 | 89200.4 | 88882.8 | 90595.9 | 89282.2 | 87920.3 |
| G | 165739.3 | 172715.1 | 176972.7 | 177222.9 | 164609.6 | 166733.8 |
| m1A | 56682.9 | 55532.2 | 59710.3 | 65959.7 | 62152.0 | 56366.1 |
| m6A | 383.6 | 321.7 | 398.2 | 367.4 | 321.2 | 288.5 |
| m5C | 1481.6 | 1362.9 | 1582.0 | 1642.4 | 1617.4 | 1314.4 |
| m3C | 974.9 | 979.0 | 1073.3 | 1075.6 | 1092.8 | 933.7 |
| hm5C | 18.2 | 21.5 | 38.8 | 39.1 | 24.9 | 22.4 |
| m3U | 127.6 | 125.7 | 151.7 | 174.0 | 136.3 | 111.6 |
| m5U | 1552.5 | 1446.6 | 1515.2 | 1740.7 | 1638.1 | 1410.5 |
| m7G | 23488.5 | 22691.8 | 23932.8 | 24554.0 | 23707.5 | 19896.3 |
| m2G | 29950.5 | 30099.4 | 32032.2 | 31657.8 | 30484.4 | 28558.6 |
