# Supplementary material for: PRMT5-Mediated ALKBH5 Methylation Promotes Colorectal Cancer Immune Evasion via Increasing CD276 Expression
Source: Research (Wash D C). 2025 Jan 8;8:0549. doi: 10.34133/research.0549 (PMC11707101; doi:10.34133/research.0549)
Supplement: Supplementary 1 — Supplementary Materials and Methods Figs. S1 to S4 Tables S1 to S6 [file research.0549.f1.zip › Supplementary Table 3.docx]

**Supplementary Table 3** Modified nucleoside ratio

| Sample | DMSO_1 | DMSO_2 | DMSO_3 | GSK595_1 | GSK595_2 | GSK595_3 |
| --- | --- | --- | --- | --- | --- | --- |
| m6A/A | 0.3932% | 0.3508% | 0.4166% | 0.3224% | 0.3388% | 0.3429% |
| m1A/A | 58.0956% | 60.5512% | 62.4761% | 57.8797% | 65.5686% | 66.9935% |
| m3U/U | 0.0816% | 0.0795% | 0.0918% | 0.0881% | 0.0922% | 0.0801% |
| m5U/U | 0.9926% | 0.9144% | 0.9172% | 0.8811% | 1.1077% | 1.0122% |
| m7G/G | 14.1719% | 13.1383% | 13.5234% | 13.8549% | 14.4022% | 11.9329% |
| m2G/G | 18.0708% | 17.4272% | 18.1001% | 17.8633% | 18.5192% | 17.1283% |
| m5C/C | 1.6943% | 1.5279% | 1.7799% | 1.8129% | 1.8115% | 1.4950% |
| hm5C/C | 0.0208% | 0.0241% | 0.0437% | 0.0432% | 0.0278% | 0.0255% |
| m3C/C | 1.1150% | 1.0975% | 1.2075% | 1.1872% | 1.2240% | 1.0620% |
