# Supplementary material for: PRMT5-Mediated ALKBH5 Methylation Promotes Colorectal Cancer Immune Evasion via Increasing CD276 Expression
Source: Research (Wash D C). 2025 Jan 8;8:0549. doi: 10.34133/research.0549 (PMC11707101; doi:10.34133/research.0549)
Supplement: Supplementary 1 — Supplementary Materials and Methods Figs. S1 to S4 Tables S1 to S6 [file research.0549.f1.zip › Supplementary Table 4.docx]

**Supplementary Table 4** Relationship between meALKBH5 expression and clinicopathological features of CRC patients

| Variables | All patients | meALKBH5 expression | | *P*-value^*^ |
| --- | --- | --- | --- | --- |
|  |  | Low (%) | High (%) |  |
| All cases | 212 | 63 (30) | 149 (70) |  |
| Age |  |  |  | 0.725 |
| ＜60 years | 67 | 21 (31) | 46 (69) |  |
| ≥60 years | 145 | 42 (29) | 103 (71) |  |
|  |  |  |  |  |
| Gender |  |  |  | 0.937 |
| Males | 85 | 25 (29) | 60 (71) |  |
| Females | 127 | 38 (30) | 89 (70) |  |
|  |  |  |  |  |
| TNM stage |  |  |  | ＜0.0001 |
| I | 21 | 19 (90) | 2 (10) |  |
| II | 83 | 43 (52) | 40 (48) |  |
| III | 97 | 0 (0) | 97(100) |  |
| IV | 11 | 1 (9) | 10 (91) |  |
|  |  |  |  |  |
| Depth of invasion |  |  |  | ＜0.0001 |
| T1/T2 | 21 | 19 (90) | 2 (10) |  |
| T3/T4 | 191 | 54 (28) | 137(72) |  |
|  |  |  |  |  |
| Metastasis |  |  |  | 0.008 |
| M0 | 201 | 101 (50) | 100 (50) |  |
| M1 | 11 | 1 (9) | 10 (91) |  |

*P*-value^*^ measured by Pearson’s Chi-Squared test.
