# Supplementary material for: PRMT5-Mediated ALKBH5 Methylation Promotes Colorectal Cancer Immune Evasion via Increasing CD276 Expression
Source: Research (Wash D C). 2025 Jan 8;8:0549. doi: 10.34133/research.0549 (PMC11707101; doi:10.34133/research.0549)
Supplement: Supplementary 1 — Supplementary Materials and Methods Figs. S1 to S4 Tables S1 to S6 [file research.0549.f1.zip › Supplementary Table 5.docx]

**Supplementary Table 5** Univariate Cox regression analysis of meALKBH5 expression and clinicopathologic variables predicting the survival of CRC patients

| **Variables** | **Overall Survival** | | **Disease Free Survival** | |
| --- | --- | --- | --- | --- |
|  | **HR (95%CI)** | ***P*** | **HR (95%CI)** | ***P*** |
| meALKBH5 | 4.604 (2.512–8.440) | ＜0.001 | 4.694 (1.607–13.713) | 0.005 |
| Age | 1.545 (0.989–2.415) | 0.056 | 3.116 (1.073–9.047) | 0.037 |
| Gender | 1.484 (1.005–2.191) | 0.047 | 1.847 (0.855–3.989) | 0.118 |
| Tumor diameter | 2.985 (1.957–4.551) | ＜0.001 | 7.405 (2.784–19.694) | ＜0.001 |
| Differentiation | 0.267 (0.180–0.395) | ＜0.001 | 0.134 (0.061–0.292) | ＜0.001 |
| TNM stage | 3.068 (2.003–4.704) | ＜0.001 | 5.629 (2.254–14.062) | ＜0.001 |
| Depth of invasion | 1.989 (1.130–3.501) | 0.017 | 2.847 (0.854–9.494) | 0.089 |
| LNM | 1.743 (1.181–2.571) | 0.005 | 1.969 (0.913–4.249) | 0.084 |
| Metastasis | 3.394 (1.243–9.271) | 0.017 | 6.370 (1.486–27.301) | 0.013 |

Abbreviations: HR: Hazard Ratio; CI: Confidence Interval; *P*: *P*-value; LNM: Lymph Node Metastasis.

Variables: meALKBH5: High *vs* Low; Age: ≥60(years) *vs* ＜60(years); Gender: Male *vs* Female; Tumor diameter: ＞5(cm) *vs* ≤5(cm); Differentiation: Moderate/High *vs* Poor; MSI: High *vs* Low; TNM stage: III/IV *vs* I/II; Depth of invasion: T3/T4 *vs* T1/T2; LNM: N1/N2 *vs* N0; Metastasis: M1 *vs* M0.

Univariate Cox regression *P*-value＜0.05 is considered to be statistically significant.
