# Supplementary material for: PRMT5-Mediated ALKBH5 Methylation Promotes Colorectal Cancer Immune Evasion via Increasing CD276 Expression
Source: Research (Wash D C). 2025 Jan 8;8:0549. doi: 10.34133/research.0549 (PMC11707101; doi:10.34133/research.0549)
Supplement: Supplementary 1 — Supplementary Materials and Methods Figs. S1 to S4 Tables S1 to S6 [file research.0549.f1.zip › Supplementary Table 6.docx]

**Supplementary Table 6** Multivariate Cox regression analysis of meALKBH5 expression and clinicopathologic variables predicting the survival of CRC patients

| **Variables** | **Overall Survival** | | **Disease Free Survival** | |
| --- | --- | --- | --- | --- |
|  | **HR (95%CI)** | ***P*** | **HR (95%CI)** | ***P*** |
| meALKBH5 | 2.588 (1.174-5.707) | 0.018 | 1.612 (1.085-2.394) | 0.048 |
| Differentiation | 0.377 (0.234-0.608) | <0.001 | 0.690 (0375-1.270) | 0.233 |
| TNM stage | 1.316 (0.704-2.459) | 0.389 | 1.536 (0.687-3.435) | 0.296 |
| Depth of invasion | 1.101 (0.596-2.035) | 0.759 | 1.689 (0.673-1.270) | 0.265 |
| LNM | 1.503 (0.898-2.515) | 0.121 | 1.900 (0.939-3.843) | 0.074 |
| Metastasis | 2.050 (0.737-5.700) | 0.169 | 1.566 (0.357-6.879) | 0.552 |

Abbreviations: HR: Hazard Ratio; CI: Confidence Interval; *P*: *P*-value; LNM: Lymph Node Metastasis.

Variables: meALKBH5: High *vs* Low; Differentiation: Moderate/High *vs* Poor; MSI: High *vs* Low; TNM stage: III/IV *vs* I/II; Depth of invasion: T3/T4 *vs* T1/T2; LNM: N1/N2 *vs* N0; Metastasis: M1 *vs* M0.

Multivariate Cox regression *P*-value＜0.05 is considered to be statistically significant.
